# Blood metabolomic fingerprint is distinct in healthy coronary and in stenosing or microvascular ischemic heart disease

**DOI:** 10.1186/s12967-017-1215-7

**Published:** 2017-05-23

**Authors:** Martino Deidda, Cristina Piras, Christian Cadeddu Dessalvi, Damiana Congia, Emanuela Locci, Federica Ascedu, Gianfranco De Candia, Mauro Cadeddu, Giorgio Lai, Raimondo Pirisi, Luigi Atzori, Giuseppe Mercuro

**Affiliations:** 1 Department of Medical Sciences and Public Health, University of Cagliari, Monserrato, Italy; 2 Department of Biomedical Sciences, University of Cagliari, Monserrato, Italy; 3 Cardiology Complex Unit, Azienda Osperaliera Brotzu, Cagliari, Italy; 4 Cath Lab, Azienda Ospedaliero-Universitaria di Cagliari, Cagliari, Italy; 5 Department of Medical Sciences and Public Health, University of Cagliari, Asse didattico Medicina, Cittadella Universitaria, SS Sestu KM 0.700, 09042 Monserrato, Italy

**Keywords:** Metabolomics, Atherosclerosis, Endothelial function, Coronary artery

## Abstract

**Background:**

The endothelium is a key variable in the pathogenesis of atherosclerosis and its complications, particularly coronary artery disease (CAD). Current evidence suggests that the endothelial status can be regarded as an integrated index of individual atherogenic and anti-atherogenic properties, and that the interaction between circulating factors and the arterial wall might be critical for atherogenesis. In organism-level investigations, a functional view is provided by metabolomics, the study of the metabolic profile of small molecules. We sought to verify whether metabolomic analysis can reveal the presence of coronary microenvironment peculiarities associated with distinct manifestations of CAD.

**Methods:**

Thirty-two coronary blood samples were analyzed using ^1^H-NMR-based metabolomics. Samples collected from patients with evidence of myocardial ischemia formed the case group, and were further divided into the stenotic-disease (SD) group (N = 13) and absence of stenosis (microvascular disease; “Micro”) group (N = 8); specimens of patients presenting no evidence of ischemic heart disease (dilated cardiomyopathy, valvular diseases) constituted the control group (N = 11).

**Results:**

Application of an orthogonal partial least squares discriminant analysis (OPLS-DA) model to the entire dataset clearly separated the samples into 3 groups, indicating 3 distinct metabolic fingerprints. Relative to control-group members, Micro patients showed a higher content of 2-hydroxybutirate, alanine, leucine, isoleucine, and *N*-acetyl groups and lower levels of creatine/phosphocreatine, creatinine, and glucose, whereas SD patients showed higher levels of 3-hydroxybutirate and acetate and a lower content of 2-hydroxybutirate. Moreover, relative to SD patients, Micro patients showed higher levels of 2-hydroxybutirate, alanine, leucine, and *N*-acetyl groups and lower levels of 3-hydroxybutirate and acetate.

**Conclusions:**

Specific coronary microenvironments are likely associated with distinct development and pathological expression of CAD.

**Electronic supplementary material:**

The online version of this article (doi:10.1186/s12967-017-1215-7) contains supplementary material, which is available to authorized users.

## Background

Atherosclerosis is currently the leading cause of death and disability in developing countries and is mainly expressed in the form of coronary artery disease (CAD), which is associated with high morbidity and mortality [1, 2].

Although systemic cardiovascular risk factors for atherosclerosis (hyperlipidemia, diabetes mellitus, smoking and hypertension) have been identified [3–9], a wide series of researches suggest that the local microenvironment, that comprehends arterial mechanics, matrix remodelling and lipid deposition, plays a key role in regulating the susceptibility to plaque development and progression, regulating the function of endothelial cells. Moreover, these microenvironmental stimuli are capable of modulate other aspects of the microenvironment through collective adaptation [10]. All these events induce a series of biochemical reactions that generate a wide and complex interplay between metabolites absorbed from or released in blood [11], thus modifying also the vascular microenvironment.

In this context, the omics approach could represent an innovative method for comprehensively investigating the molecular basis of CAD pathogenesis.

During the past decade, both animal and human studies have enabled the rapid development of metabolomics. By combining targeted and non-targeted approaches, metabolomic analysis has identified small-molecule metabolite profiles of diverse cardiovascular risk factors and diseases [11–13].

On this basis, our hypothesis was that (i) despite similar cardiovascular risk factors, coronary different microenvironments, generated by interaction between inherited and acquired factors, could generate distinct manifestations of CAD and (ii) these microenvironmental differences might be investigated by metabolomics analysis.

## Methods

### Study participants

The study was approved by the Institutional Ethics Committee (Azienda Ospedaliero-Universitaria of Cagliari) and was performed in accordance with the Declaration of Helsinki.

We consecutively enrolled patients requiring coronary angiography among those screened in our clinic; they were informed of the purpose and methodology of the study and their written consent was obtained prior to inclusion. They underwent a complete cardiovascular assessment, which included medical history evaluation, physical examination, blood pressure measurement, 12-lead electrocardiogram and echocardiogram, in accordance with the American Society of Echocardiography/European Association of Cardiovascular Imaging guidelines [14]. The patients were also submitted to a physical or pharmacological stress test, in order to confirm/exclude the diagnosis of angina. The patients with myocardial ischemia confirmed by provocative testing formed the case group, and were further subdivided, on the basis of coronary angiography, in patients with stenotic disease (SD group; N = 13) and without stenosis (microvascular disease, “Micro” group; N = 8). Patients with indications for coronary angiography different from angina/myocardial infarction (i.e. dilated cardiomyopathy, valvular disease), normal coronary angiogram and no evidence of atherosclerotic lesions in other vascular district constituted the control group (N = 11). In total, thirty-two coronary blood samples were collected. Table 1 lists the full inclusion and exclusion criteria.

**Table 1:** Study population: inclusion and exclusion criteria

Inclusion criteria	Exclusion criteria
Case groups	
Age >18 years Stable Angina diagnosed in accordance with ESC guidelines Stress test positive for ischemia	Recent PTCA (1 month for BMS, 1 year for DES) Inheritable metabolic disorders Malignancies Autoimmune diseases Hepatic (transaminases ≥2 the upper limits of normal) or renal (creatinine ≥2.0 mg/dL) dysfunction
Control groups	
Age >18 years Indications for coronary angiography different from angina/myocardial infarction	Previous or present diagnosis of CAD Coronary, carotid or peripheral arteries ATS Moderate/severe hypertension or hypertension-induced organ damage (i.e. Left ventricular hypertrophy) Inheritable metabolic disorders Malignancies Autoimmune diseases Hepatic (transaminases ≥2 the upper limits of normal) or renal (creatinine ≥2.0 mg/dL) dysfunction

In Table 2, the anthropometric and clinical data of the patients are summarized; no significant differences were present among the 3 groups.

**Table 2:** Anthropometric and clinical data of enrolled patients

	SD group	Micro group	Control group
Age (years)	65.5 ± 8.08	65.8 ± 7.95	60.8 ± 9.00
M/F (%)	61/39	50/50	55/45
Height (m)	1.63 ± 0.07	1.54 ± 0.09	1.68 ± 0.1
Weight (kg)	70.0 ± 8.9	62.4 ± 5.4	90.1 ± 29.9
BMI (kg/m^2^)	26.4 ± 3.83	26.21 ± 2.96	31.2 ± 5.7
Serum creatinine (mg/dL)	1.38 ± 0.92	0.93 ± 0.11	1.08 ± 0.3
Diabetes	4	1	0
Hypertension	6	2	1
Hypercholesterolemia	3	3	1
Smoking	2	1	1
Upper-extremity artery disease	1	1	0
Carotid artery disease	1	0	0

### Coronary angiography

All participants underwent a coronary angiography, during which a blood sample was collected after a good insertion of the diagnostic catheter into the coronary ostium and before performing any procedure on the vessel. The examinations were performed in accordance with specific published guidelines [15]. Arterial blood was aspirated through the catheter by a 5 mL syringe, stored in heparinized tube and maintained at 4 °C until it was centrifuged at 4000 rpm for 15′ and aliquoted in 800 μL cuvettes. Plasma samples were then stored at −80 °C. The contrast medium used was iomeprol (Iomeron^®^, Bracco U.K. Ltd).

### Plasma sample preparation and ^1^H-NMR spectroscopy

Each plasma sample was thawed and centrifuged at 4500 rpm at 4 °C for 10 min, and then 800 μL of the plasma was dissolved in 2.4 mL of a chloroform/methanol mixture (1:1, v/v) and 350 mL of H_2_O; the solution was centrifuged at 4500 rpm at 4 °C for 30 min. The resulting ~1 mL of the hydrophilic phase, which contained the low-molecular-weight water-soluble components, was separated from the lipophilic phase, dried using a vacuum concentrator (Eppendorf), and stored at −80 °C. Before NMR analysis, the dried hydrophilic plasma extracts were re-dissolved in 700 μL of D_2_O (99.8%, Cambridge Isotope Laboratories Inc., Andover, MA, USA) and a 630-μL aliquot was transferred into 5-mm-O.D NMR tubes, to which 70 μL of TSP [3-(trimethylsilyl)propanoic acid] was added as the internal standard (98 atom% D, Sigma-Aldrich, Milan, Italy).

^1^H-NMR experiments were conducted using a Varian UNITY INOVA 500 spectrometer operating at 499,839 MHz for protons and equipped with a 5-mm double-resonance probe (Agilent Technologies, Santa Clara, CA, USA). ^1^H-NMR spectra were acquired at 300 K with a spectral width of 6000 Hz, a 90° pulse, an acquisition time of 2 s, a relaxation delay of 2 s, and 256 scans. A presaturation sequence was used to suppress the residual H_2_O signal by applying low-power selective irradiation at the H_2_O frequency during the recycle delay. Moreover, ^1^H–^1^H COSY 2D NMR spectra were acquired. The experiment was performed by using a spectral width of 6000 Hz in both dimensions, 2048 data points, and 512 increments with 32 transients per increment. The COSY experiment was conducted using a gradient-selected coherence transfer pathway (gCOSY).

### Data processing and statistical analysis

^1^H-NMR spectra were imported into ACDlab Processor Academic Edition (Advanced Chemistry Development, 12.01, 2010) and pre-processed with a line-broadening of 0.3 Hz, zero-filled to 64 K, and Fourier transformed. Each spectrum was manually phased and baseline-corrected. Chemical shifts were referred to the TSP single resonance at 0.00 ppm. The spectra were then imported into MATLAB (R2013b; Mathworks, Inc., Natick, MA, USA), where the spectra were aligned using Interval Correlation Optimized Shifting (*i*coshift) [16] to correct for small peak misalignments of certain metabolites. The spectral region between 4.74 and 4.94 ppm was excluded from the analysis in order to remove the effect of variations in the presaturation of the residual water resonance. Lastly, the spectral dataset was normalized to the total area in order to minimize the effects of variable concentration among distinct samples, and then imported into the SIMCA-P+ program (Version 13.0, Umetrics, Malmö, Sweden) for multivariate statistical analysis. Data were Pareto scaled and subjected to Hotelling’s T2 and DModX tests in order to verify the presence of potential outliers. Orthogonal partial least squares discriminant analysis (OPLS-DA), a supervised method, was used to build models and identify the variables that can discriminate between patients and controls. Since OPLS-DA can lead to over-fit models, particularly with few samples as in the present study, we verified the model quality by performing a sevenfold cross-validation and permutation test 400 times on the corresponding partial least square discriminant analysis (PLS-DA) model. Overfitting and predictive ability of the models were assessed using R^2^ and Q^2^ values. To evaluate the differences in metabolites between the 3 groups, a parametric Student’s test (with *P* values corrected with the Bonferroni method) was performed on the relative concentration of the NMR signals determined using Chenomx NMR suite 7.1 (Chenomx Inc., Edmonton, AB, Canada). The receiver operating-characteristic (ROC) curve was analyzed with sensitivity versus 1—specificity, and the area under the curve (AUC) was calculated using the free software package ROCCET: ROC Curve Explorer and Tester (http://metaboanalyst.ca/faces/Secure/upload/RocUploadView.xhtml) [17]. An AUC >0.8 indicates a test exhibiting good discrimination between controls and patients.

## Results

### Coronary angiography

In accordance with the inclusion/exclusion criteria, the control-group patients showed no coronary stenosis or alterations in the TIMI angiographic flow grades. The patients in the Micro group exhibited no coronary atherosclerotic lesions, but presented a low (1–2) TIMI flow grade and, in one case, coronary spasm induced by catheter irrigation. By contrast, the angiographic findings revealed that the SD group included 2 patients with significant (>75% diameter stenosis or FFR <80%) monovascular disease, 6 with bivascular disease, and 5 with left main/trivascular disease.

### Metabolomic analysis

**Fig. 1:**
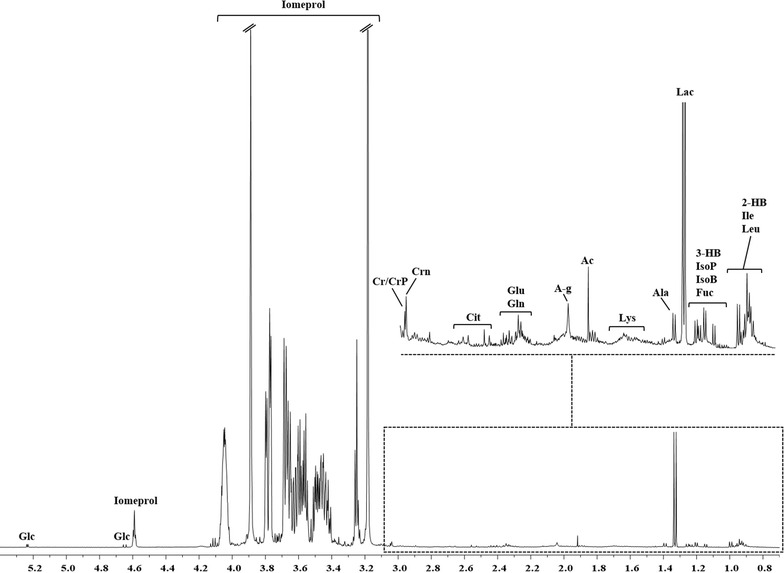
Resonance assignment of a representative ^1^H-NMR spectrum of a plasma extract. *Peaks* 2-hydroxybutyrate (2-HB), 3-hydroxybutyrate (3-HB), acetate (Ac), alanine (Ala), citrate (Cit), creatine/phosphocreatine (Cr/CrP), creatinine (Crn), fucose (Fuc), glucose (Glc), glutamate (Glu), glutamine (Gln), isobutyrate (IsoB), isoleucine (Ile), isopropanol (IsoP), lactate (Lac), leucine (Leu), lysine (Lys), and *N*-acetyl group (A-g)

**Fig. 2:**
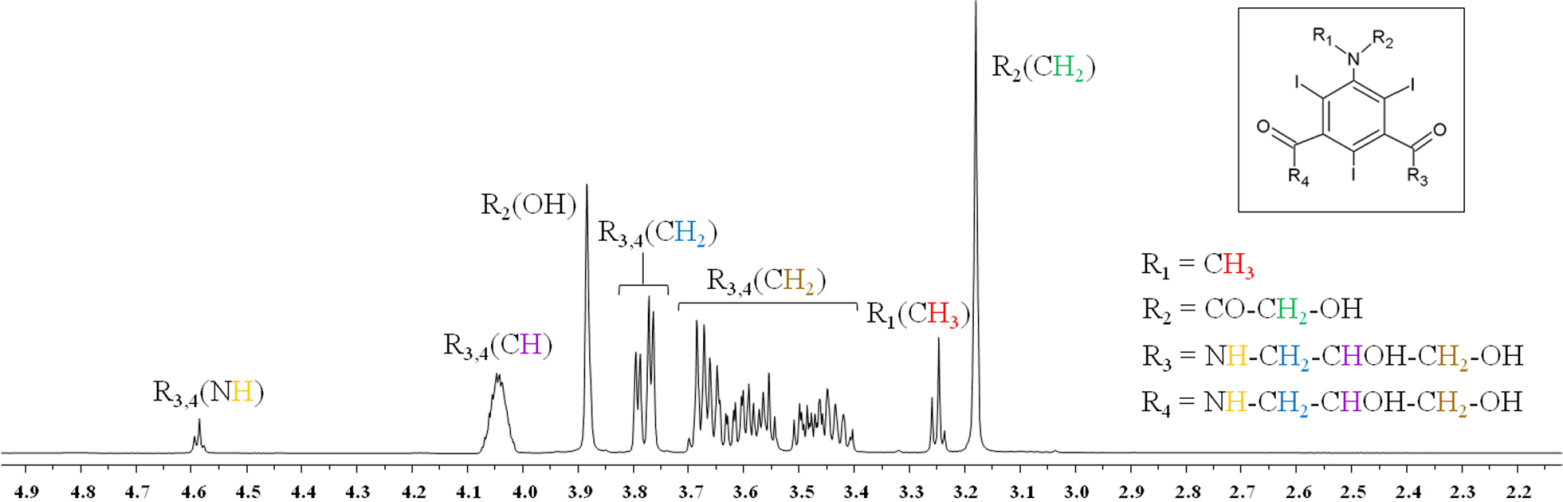
^1^H-NMR Spectrum of Iomeprol. Iomeprol: commercial name, Iomeron 350; IUPAC name,* N*,*N*ʹ-bis-(2,3-dihydroxypropyl)-5-[(hydroxyacetyl)methylamino]-2,4,6-tri-iodo-1,3-benzenedicarboxamide)

The metabolites were identified with the aid of the literature and databases such as Human Metabolome Database (HMDB, http://www.hmdb.ca) and the 500-MHz library from Chenomx NMR suite 7.1. The attribution of the major peaks of the plasma samples are shown in Fig. 1, whereas the chemical shifts of all metabolites are summarized in Table 3. In addition to the signals due to the metabolites, such as amino acids, sugars, and organic acids from the plasma, the spectra displayed a series of extremely intense resonances in the region between 3.1 and 4.2 ppm; these do not belong to the physiological metabolic profile of the plasma but to the contrast agent employed in the standard procedure of arterial blood collection. Both 1D and 2D NMR experiments were conducted on a solution of the contrast agent (Fig. 2; Additional file 1: Figure S1), which allowed discrimination of its characteristic resonances, and these were then excluded from the statistical analysis performed on the plasma spectra.

**Table 3:** Proton chemical shifts of metabolites identified in 500-MHz ^1^H-NMR spectra of plasma samples

Metabolites	Group	^1^H ppm^a^	^1^H Multiplicity
2-Hydroxybutyrate	CH_3_	0.90	t
	CH_2_	1.68	m
	CH	4.02	dd
3-Hydroxyburyrate	CH_3_	1.20	d
	CH_2_	2.35	dd
	CH	4.12	m
Acetate	CH_3_	1.91	s
Alanine	CH_3_	1.37	d
Citrate	γCH_2_	2.55	d
	γ’CH_2_	2.68	d
Creatine/PCreatine	N-CH_3_	3.04	s
Creatinine	N-CH_3_	3.03	s
Fucose	αCH_3_	1.22	d
	βCH_3_	1.25	d
α-Glucose	C_1_H	5.23	d
β-Glucose	C_1_H	4.64	d
Glutamate	βCH_2_	2.12	m
	γCH_2_	2.34	m
Glutamine	βCH_2_	2.14	m
	γCH_2_	2.43	m
Isobutyrate	CH_3_	1.20	d
Isopropanol	CH_3_	1.12	d
Isoleucine	βCH	1.98	m
	γCH	1.46	m
	γ’CH	1.25	m
	γ’CH_3_	0.99	d
	δCH_3_	0.93	t
Lactate	βCH_3_	1.32	d
	αCH	4.10	q
	εCH_2_	3.02	t
Leucine	βCH_2_	1.72	m
	γCH	1.76	m
	δCH_3_	0.94	d
Lysine	βCH_2_	1.90	m
	γCH_2_	1.46	m
	δCH_2_	1.72	m
*N*-acetyl group	CH_3_	2.06	s

Multiplicity definitions: *s* singlet, *d* doublet, *t* triplet, *q* quartet, *dd* doublet of doublets, *m* multiplet

^a^ ^1^H chemical shifts reported with respect to TSP signal (0.00 ppm)

**Fig. 3:**
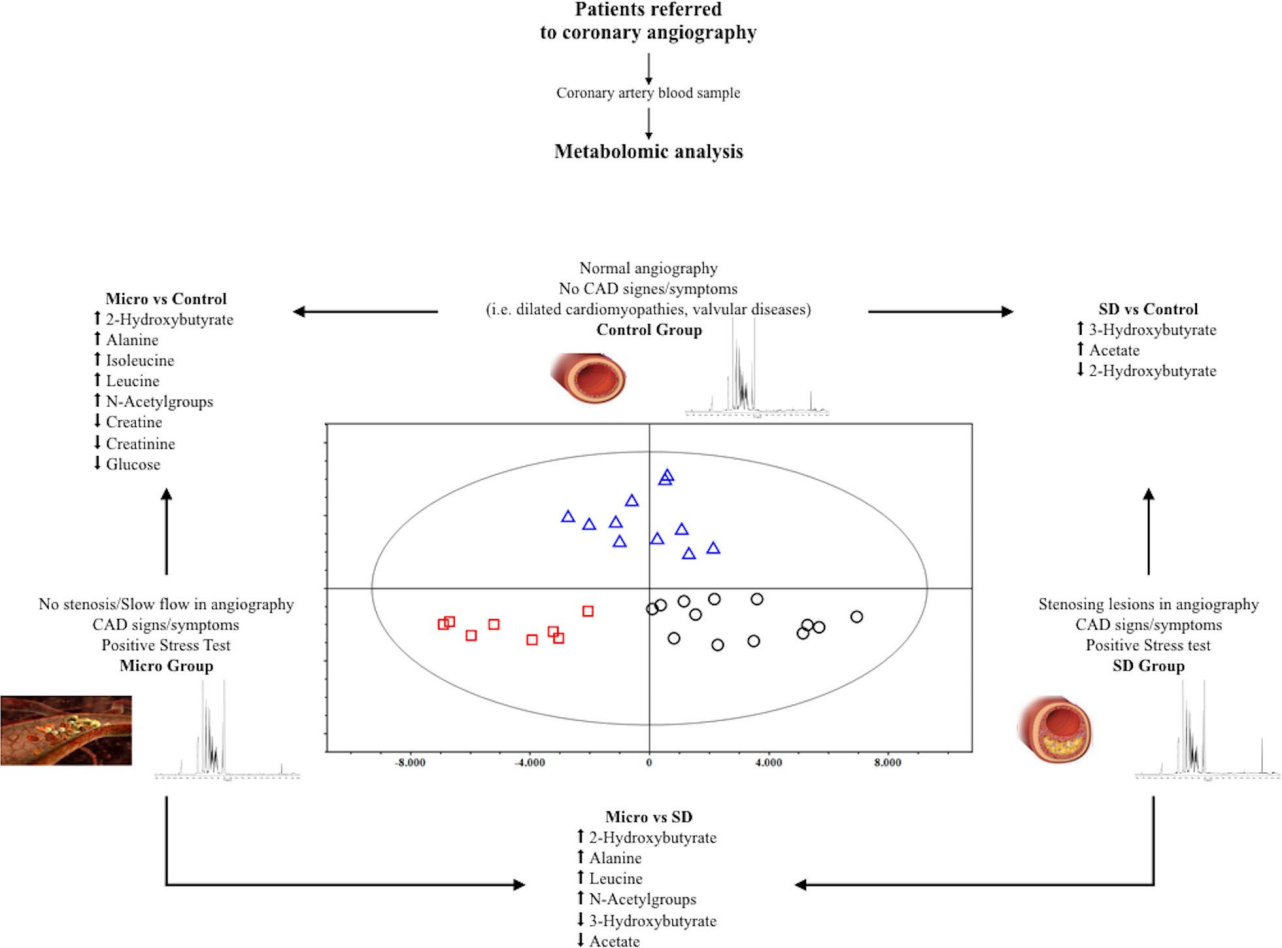
OPLS-DA score plots of ^1^H-NMR spectra of plasma samples. *Groups* control (*triangles*), Micro (*boxes*), and SD (*circles*)

**Fig. 4:**
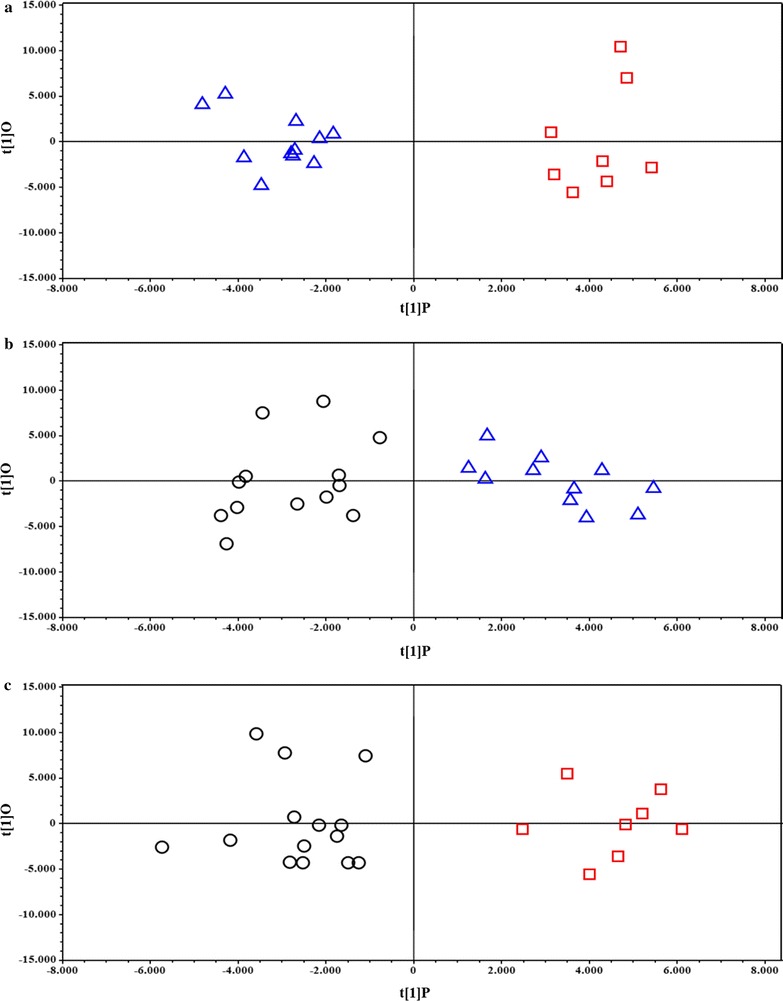
OPLS-DA Score plots derived from pairwise comparison of ^1^H-NMR spectra of plasma samples. Comparisons between groups: **a** Control (*triangles*) vs Micro (*boxes*), **b** Control (*triangles*) vs SD (*circles*), and **c** Micro (*box*) vs SD (*circle*)

**Fig. 5:**
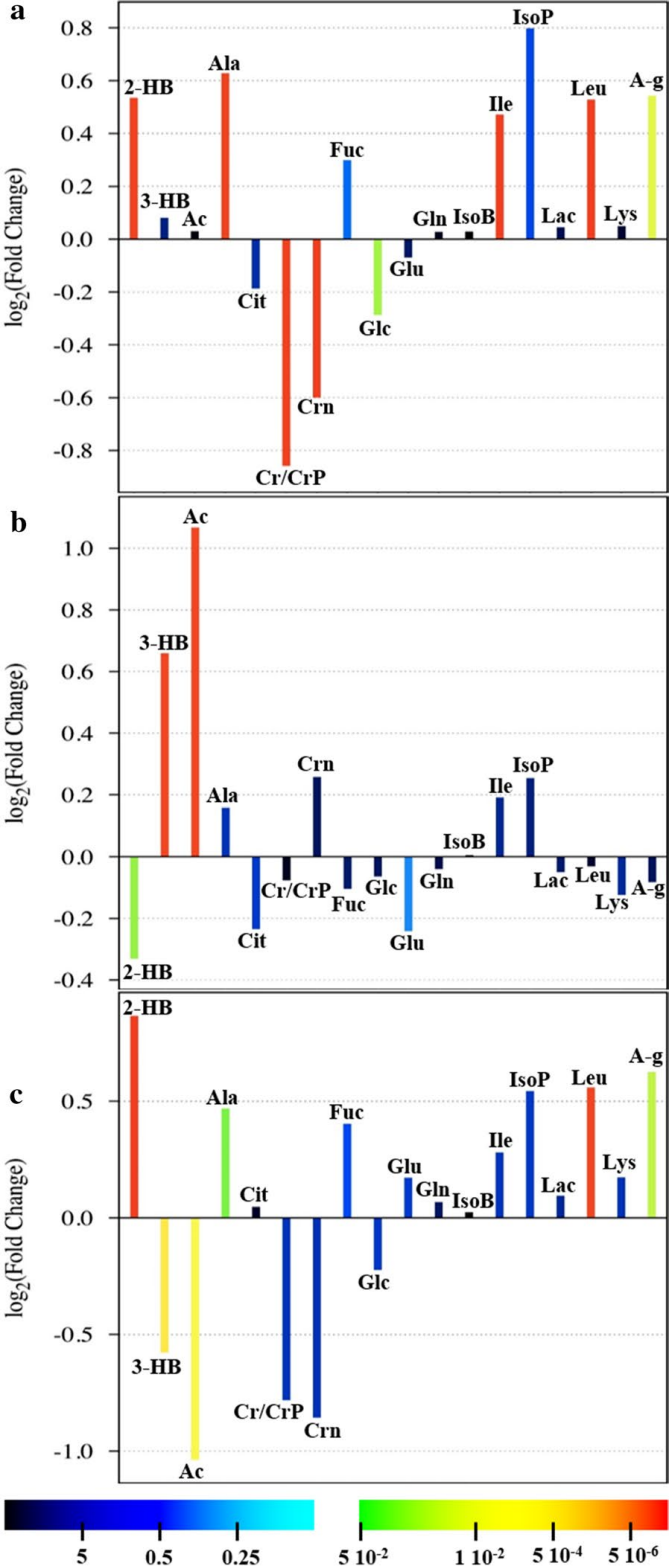
Fold-change plots color-coded based on corresponding *P* values (Corrected with Bonferroni Method) Pairwise comparisons between groups: **a** Micro vs Control, **b** SD vs Control, and **c** Micro vs SD. *Metabolite abbreviations* 2-hydroxybutyrate (2-HB), 3-hydroxybutyrate (3-HB), acetate (Ac), alanine (Ala), citrate (Cit), creatine/phosphocreatine (Cr/CrP), creatinine (Crn), fucose (Fuc), glucose (Glc), glutamate (Glu), glutamine (Gln), isobutyrate (IsoB), isoleucine (Ile), isopropanol (IsoP), lactate (Lac), leucine (Leu), lysine (Lys), *N*-acetyl group (A-g)

First, an OPLS-DA model was applied to the entire dataset; the results obtained (Fig. 3) showed that the samples were clearly separated into 3 groups, which indicated that the controls and the Micro and SD patients presented highly distinct metabolic profiles. The OPLS-DA model was established using 2 predictive components and one orthogonal component, and showed good values of R^2^Y and Q^2^ (Table 4). To validate the OPLS-DA model, a permutation test on the corresponding PLS-DA model was performed using the same number of components. The results (Table 4) clearly indicated that the OPLS-DA model was statistically valid. Three separate OPLS-DA models were built using the same 3 groups of samples—Control, Micro, and SD—but only 2 groups were compared in each case. The results of these pairwise comparisons (Fig. 4) enabled improved assessment and identification of the metabolites that were responsible for the separation between the distinct groups. The parameters for all OPLS-DA models are summarized in Table 4. In addition to the permutation test, a cross-validated score plot for the same OPLS-DA models was created (Additional file 2: Figure S2), because an external test dataset was not present. In the cross-validated score plot of SD versus Control, 2 SD and 2 Control samples were misclassified, whereas all the other samples were accurately classified within their groups. The metabolites primarily responsible for group separation were finally identified by building a V-plot for each OPLS-DA model (Additional file 3: Figure S3). The V-plot reveals the contribution of each variable to the predictive component, matching the correlation coefficients p(corr) and VIP values, and the VIP value is used to evaluate the variables’ relative importance. Here, variables (metabolites) with a VIP score of >1 were considered and their relative concentration was determined using Chenomx NMR suite 7.1, and the new dataset thus obtained was normalized and subjected to Student’s test and the results were corrected with the Bonferroni method. The “fold-change” was calculated for each of these variables and its magnitude was color-coded based on the corresponding *P* value from the Student’s test (Fig. 5). Several metabolites were found to change significantly among the 3 groups of samples. With respect to control patients, a higher content of 2-hydroxybutirate, alanine, leucine, isoleucine, *N*-acetyl groups and a lower content of creatine/phosphocreatine, creatinine, and glucose characterized the Micro patients (Fig. 5a), whereas a higher content of 3-hydroxybutirate and acetate and a lower content of 2-hydroxybutirate characterized the SD patients (Fig. 5b). Lastly, a complementary comparison between the Micro and SD patients (Fig. 5c) revealed a higher content of 2-hydroxybutirate, alanine, leucine, and *N*-acetyl groups and a lower content of 3-hydroxybutirate and acetate in the Micro group. ROC curves were created to assess the combined performance (significance) of these metabolites; the relative concentration of the metabolites allowed discrimination of the 3 groups, with the area under the ROC curve (AUC) >0.9 for each pair.

**Table 4:** Parameters for OPLS-DA models derived from ^1^H-NMR spectra of plasma samples from controls and micro and SD patients

	OPLS-DA models				Permutation*	
	Components^a^	R^2^Xcum^b^	R^2^Ycum^c^	Q^2^cum^d^	R^2^ intercept	Q^2^ intercept
Controls vs Micro vs SD	2P + 1O	0.305	0.800	0.511	0.460	−0.188
Controls vs Micro	1P + 1O	0.317	0.947	0.697	0.513	−0.161
Controls vs SD	1P + 1O	0.247	0.871	0.512	0.507	−0.147
Micro vs SD	1P + 1O	0.301	0.895	0.673	0.425	−0.144

* R^2^ and Q^2^ intercept values are indicative of a valid model. The permutation test was evaluated on the corresponding partial least square discriminant analysis (PLS-DA) model

^a^ Number of predictive and orthogonal components used to create the statistical models

^b,c^ R^2^X and R^2^Y indicate the cumulative explained fraction of the variation of the X block and Y block for the extracted components

^d^ Q^2^cum values indicate the cumulative predicted fraction of the variation of the Y block for the extracted components

## Discussion

We conducted a pathophysiological study to identify possible metabolic differences in the microenvironment of coronary arteries, normal or suffering from both stenotic and microvascular disease, using metabolomics, an innovative and highly sensitive method.

For this purpose, we conducted ^1^H-NMR experiments on blood samples obtained from coronary artery of all enrolled case-group and control patients. We obtained the following results: (1) the OPLS-DA model used here separated the samples into 3 groups, which indicated that, given comparable cardiovascular risk factors, the controls and the Micro and SD patients were characterized by markedly different metabolic profiles. (2) ROC curves, created to evaluate the importance of the relative concentration of detected metabolites, confirmed the identification of the 3 distinct biochemical fingerprints, one belonging to healthy coronary arteries, the other 2 underlying the stenotic and microvascular manifestations of CAD. (3) Pairwise comparisons used to identify the metabolites responsible for the separation among groups revealed that relative to the control group, the Micro group showed higher levels of 2-hydroxybutyrate, alanine, leucine, isoleucine, and *N*-acetyl groups and lower levels creatine/phosphocreatine, creatinine, and glucose, whereas the SD group showed higher levels of 3-hydroxybutyrate and acetate and lower levels of 2-hydroxybutirate; furthermore, the levels of 2-hydroxybutyrate, alanine, leucine, and *N*-acetyl groups were higher and those of 3-hydroxybutyrate and acetate lower in the Micro group than in the SD group.

Several studies on the pathophysiological mechanisms involved in atherogenesis have shown that alteration in endothelial function is a key event in the development of this pathology and that it plays a major role in plaque progression and complication [18–21]. By contrast, with regard to the possibility of using metabolomics for detecting the presence of CAD and defining its severity, previous studies have obtained divergent results, first supporting [22] and then refuting [23] the likelihood of this approach being employed effectively.

However, the prevalence of an interest in diagnostics diverted the attention from the endothelium as the substrate of atherogenesis.

The investigation of endothelial metabolic responses is critical: the recognized risk factors for atherosclerosis, although typically systemic, necessarily require an alteration of endothelial properties to change from a potential threat to a pathological lesion [10, 18–20].

As stated in the Introduction section, the action of neural, hormonal, paracrine or cytokine signaling directly or indirectly modifies cellular metabolic pathways [11, 24]. Furthermore, it was shown that even the endothelial mechanoreceptors activity can alter circulating metabolites and may be revealed by metabolomics [25].

Given all the aforementioned considerations, we designed this study to assess the coronary microenvironment and its relationship with the pattern and degree of arterial anatomical and functional impairment in patients undergoing coronary angiography. Patients with stable angina (confirmed by positive stress test) formed the 2 case groups (Micro and SD), whereas patients with non-ischemic diseases (e.g., valvular heart disease, cardiomyopathies) were enrolled as controls. According to our OPLS-DA model, the coronary artery blood of CAD patients with stenosing disease appeared different, with high specificity, from that of patients with microvascular disease. Furthermore, the metabolomic analysis differentiated both case groups from the control group of non-ischemic patients; this discrimination was further confirmed using ROC curves, which validated the OPLS-DA models in terms of sensibility and sensitivity [17].

The interaction between “macro” risk factors (e.g. diabetes, hypertension, dyslipidaemia) and intrinsic properties (e.g. genetic, epigenetic) of the local endothelial layer may be critical for atherogenesis [10, 26]. This interaction may produce a microenvironment suitable for the phenotypic translation of a specific athero-susceptibility, as previously suggested by a wide range of studies [10].

The metabolic profile provides a “functional” view of a tissue, intended as the ultimate resultant of its genes, RNAs, proteins and environmental factors (e.g. nutrition). In the case of the endothelium, it may provide evidence of a perturbated blood flow (e.g. the presence of a plaque), reflecting the endpoints of the biological processes of that tissue and the involved pathways [11, 12]. Accordingly, despite the comparable systemic risk factors, all the groups in our study showed different coronary angiography findings and a distinctive metabolic fingerprint, as confirmed by the statistical parameters reported in Table 4. The control group was distinct from both case groups (PC2, Fig. 3), whereas the latter seem to be differentiated along the PC1.

Our results suggest that the normal coronary microenvironment is considerably different from that of coronary arteries affected by both stenotic and microvascular disease. In addition, coronaries with atherosclerosis exhibit different metabolic fingerprints from each other, suggesting the involvement of distinctive pathways in these 2 atherogenic manifestations.

The different expressions or developmental stages of the same disease explained by metabolomics suggest that endothelial dysfunction, though enabled by the classic cardiovascular risk factors, is modulated by other determinants, such as genetic, nutritional and behavioral, and causes various local micro-environments, witnessing an individual sensitivity to atherosclerosis. The cluster of identified metabolites, specific for each group, as confirmed by the ROC curves, suggests the existence of multiple pathways involved in atherosclerotic development and responsible for the differences we encountered.

Coronary blood from the SD group contained higher levels of acetoacetate and 3-hydroxybutyrate but lower levels of 2-hydroxybutyrate than did the blood from both Micro and Control groups. Acetoacetate and 3-hydroxybutyrate, but not 2-hydroxybutyrate, have been associated with atherogenesis in diabetic patients [27, 28]. Specifically, Kanikarla-Marie and Jain showed that these 2 ketone bodies upregulate NADPH oxidase and thus cause, at the endothelial level, an increase in oxidative stress, ICAM-1 expression, and monocyte adhesion [28], all of which are involved in atherosclerosis development.

Relative to controls, Micro patients showed elevated levels of alanine, leucine/isoleucine, 2-hydroxybutyrate, and *N*-acetyl groups, but lower levels of creatine and creatinine. Creatine exerts anti-inflammatory effects at the endothelial level by inhibiting ICAM-1 and E-selectin expression and thereby reducing neutrophil margination. Furthermore, creatine can reduce endothelial permeability [29], and Sestili et al. have highlighted the cytoprotective effect of creatine as a radical scavenger against reactive oxygen (in particular hydroxyl radical) and nitrogen species [30].

Recently, alanine was found to be independently associated with major adverse cardiovascular events, [31] but reduced alanine levels were also reported in both apolipoprotein E-deficient mice (apolipoprotein E(−/−) mice) [32] and the plasma of patients with stable carotid atherosclerosis [33]. This dual behavior of alanine (association with cardiovascular events, but reduction in patients with atherosclerosis) was also observed in our study population: alanine levels were higher in Micro patients than in both controls and SD patients, which suggests a risk profile wherein ischemic events are not associated with atherosclerotic plaques. A similar functional behavior was also observed for leucine/isoleucine, which reduced the serum levels of SOD and GPx in hypercholesterolemic rats [34], but also positively correlated with acetoacetate and 3-hydroxybutyrate in distinguishing atherosclerotic patients from healthy controls [33].

## Conclusion

Vascular endothelial cells maintain the interface between systemic circulation and tissues and mediate several critical processes such as inflammation, coagulation, and homeostasis [10, 18–21, 26]. Our results show that the metabolic fingerprint of healthy coronary arteries is considerably different from that of atherosclerotic vessels. In addition, the metabolic profile of microvascular dysfunction is distinguishable from that of stenotic disease, despite the presence of comparable cardiovascular risk factors, thereby suggesting different underlying pathways or their different modulation. Surely, complete understanding of this metabolic interaction will require larger studies and the use of integrated (clinical, metabolomic, genomic) approaches. The data gathered from these investigations will improve our understanding of the natural history of the disease. They will help researchers identify new diagnostic tools (i.e. bedside biomarkers assays) and therapeutic targets to modulate endothelial responses and affect the progress of CAD (i.e. coronary stent eluting specific metabolites with demonstrated anti-atherogenic effects on endothelium).

### Study limitations

The small number of samples included in this study is certainly a limitation; however, the verification tests and the level of significance seem to confirm the quality of the constructed model. Thus, the involvement of the clustered metabolites in the likelihood of pathophysiologic mechanisms and the solid evidence that the identified metabolites have been associated with cardiovascular events in similar subgroups of patients are significant.

Our study does not pretend to be exhaustive or conclusive, but want to help clarify the reasons for the different individual susceptibility to the development of atherosclerosis, and stimulate further research in this direction.

## Additional files

**Additional file 1: Figure S1.** NMR 2D-COSY spectrum of Iomeprol (commercial name: Iomeron 350; IUPAC name: *N*,*N*’-bis-(2,3-dihydroxypropyl)-5-[(hydroxyacetyl)methylamino]-2,4,6-tri-iodo-1,3-benzenedicarboxamide).

**Additional file 2: Figure S2.** Cross-validated score of OPLSA-DA model derived from the pairwise comparison of ^1^H NMR spectra of plasma. For each observation, score value from the model (circle) and from the cross validation (box) are shown.

**Additional file 3: Figure S3.** V-plot with *p(corr)* and VIP values derived from the pairwise comparison of ^1^H NMR spectra of plasma in OPLS-DA models (Fig. 4 of the main paper): a) Controls versus Micro, b) Controls versus CAD, and Micro versus CAD.

## Abbreviations

AUC: area under the curve
CAD: coronary artery disease
FFR: fractional flow reserve
GPx: glutathione peroxidase
^1^H-NMR: ^1^H-nuclear magnetic resonance
ICAM: intercellular adhesion molecule
NADPH: nicotinamide adenine dinucleotide phosphate
OPLS-DA: orthogonal partial least squares discriminant analysis
PLS-DA: partial least squares discriminant analysis
ROC curve: receiver operating-characteristic curve
SD: stenotic disease
SOD: super dioxide dismutase
TDI: tissue Doppler imaging
TIMI: thrombolysis in myocardial infarction
