# Supplementary figures and images for: Blood metabolomic fingerprint is distinct in healthy coronary and in stenosing or microvascular ischemic heart disease
Source: J Transl Med. 2017 May 23;15:112. doi: 10.1186/s12967-017-1215-7 (PMC5442646; doi:10.1186/s12967-017-1215-7)

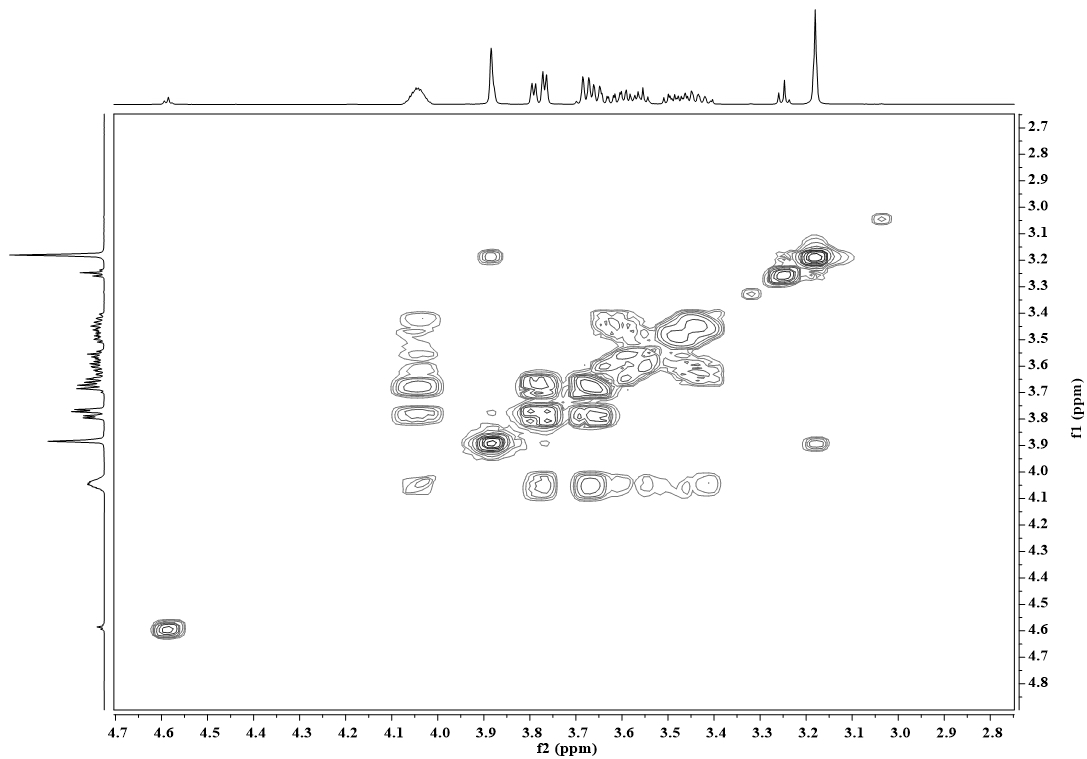

Supplement: Supplementary file 1 — Additional file 1: Figure S1. NMR 2D-COSY spectrum of Iomeprol (commercial name: Iomeron 350; IUPAC name: N,N’-bis-(2,3-dihydroxypropyl)-5-[(hydroxyacetyl)methylamino]-2,4,6-tri-iodo-1,3-benzenedicarboxamide). [file 12967_2017_1215_MOESM1_ESM.jpg]

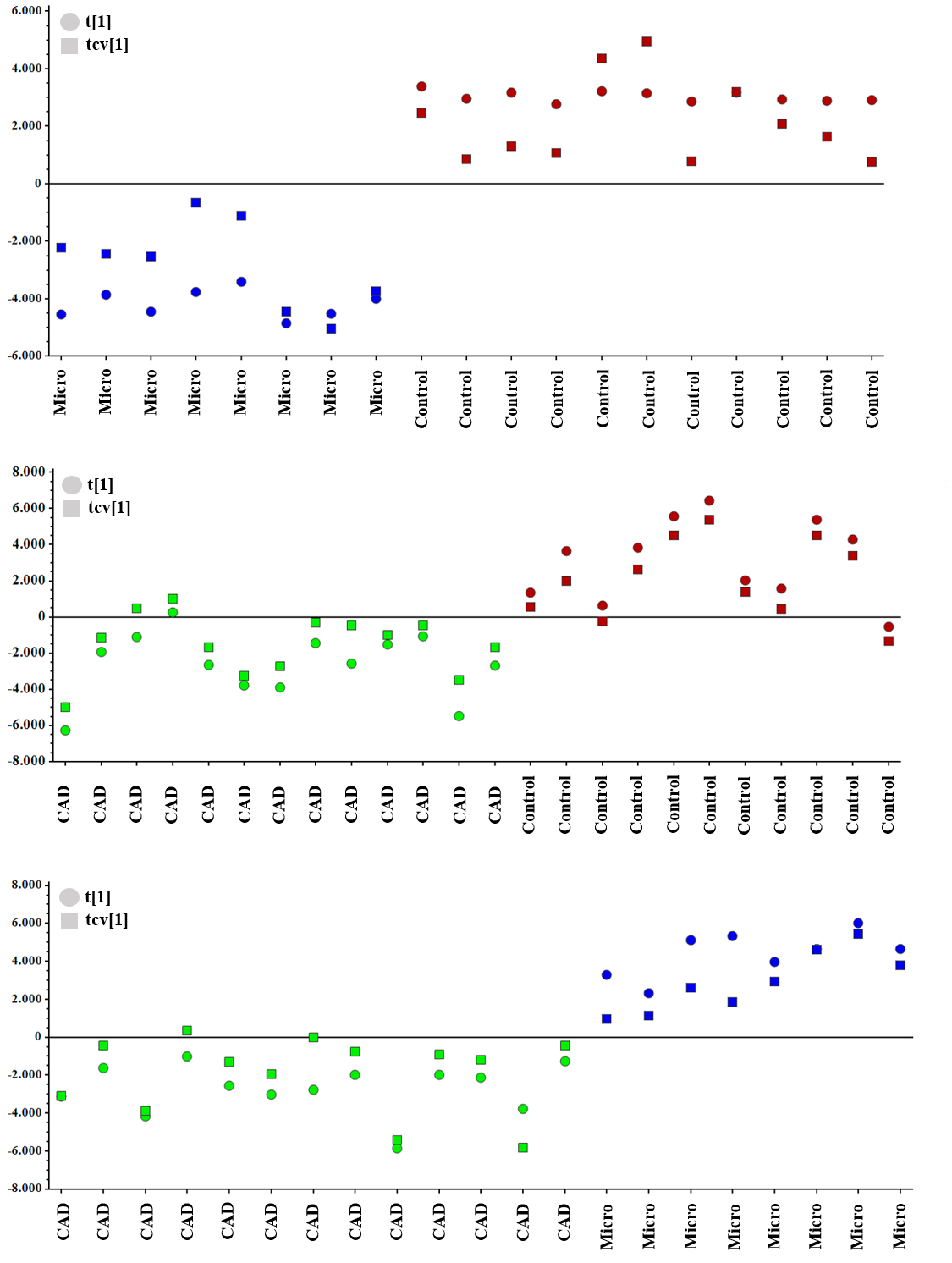

Supplement: Supplementary file 2 — Additional file 2: Figure S2. Cross-validated score of OPLSA-DA model derived from the pairwise comparison of 1H NMR spectra of plasma. For each observation, score value from the model (circle) and from the cross validation (box) are shown. [file 12967_2017_1215_MOESM2_ESM.jpg]

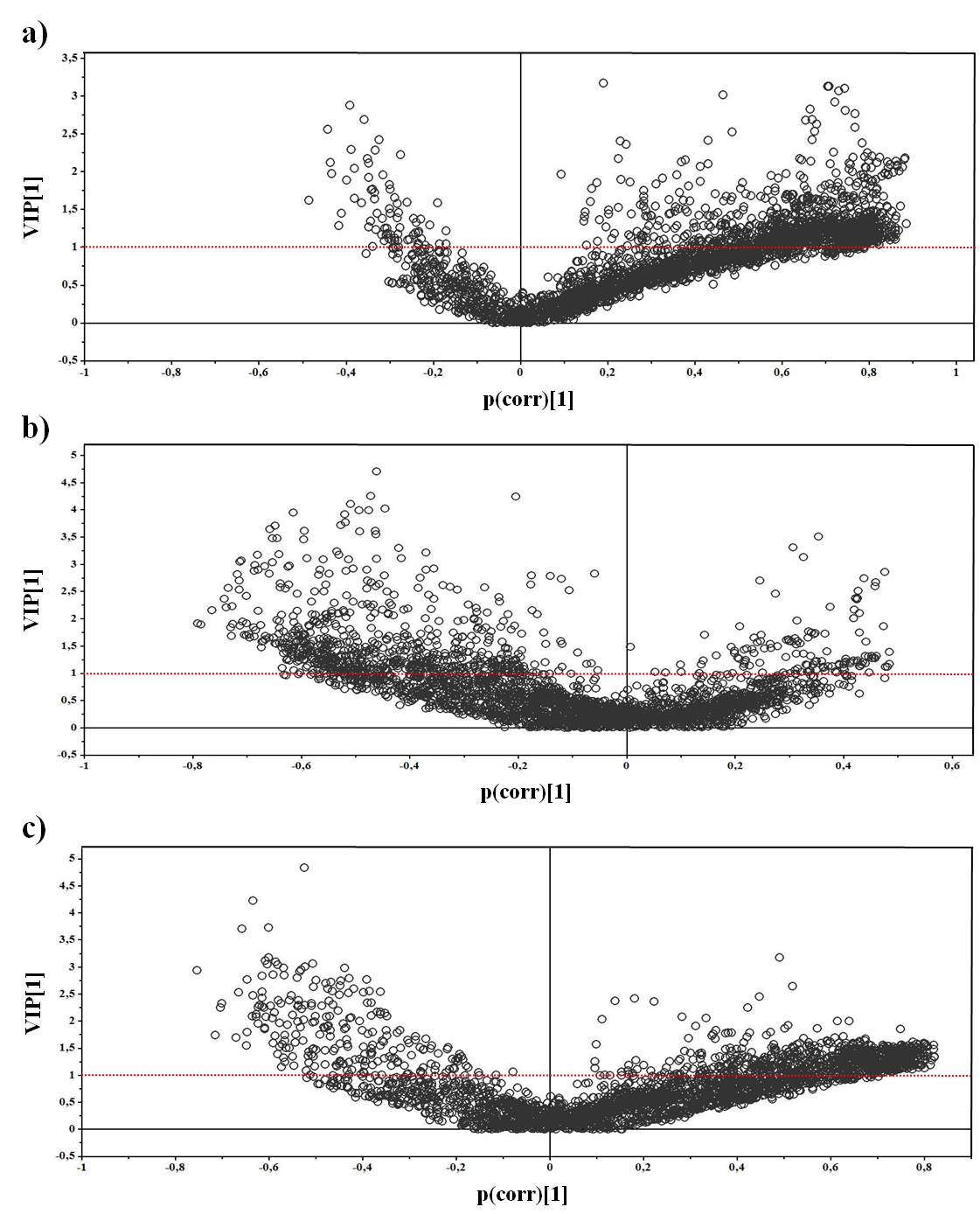

Supplement: Supplementary file 3 — Additional file 3: Figure S3. V-plot with p(corr) and VIP values derived from the pairwise comparison of 1H NMR spectra of plasma in OPLS-DA models (Fig. 4 of the main paper): a) Controls versus Micro, b) Controls versus CAD, and Micro versus CAD. [file 12967_2017_1215_MOESM3_ESM.jpg]
